# Increased anxiety symptoms in adolescents led by school phobia: a community-based study

**DOI:** 10.3389/fpsyt.2026.1773347

**Published:** 2026-02-26

**Authors:** Mengye Cao, Wenwen Shen, Fang Cheng, Wenwu Zhang, Suxian Tan, Xiulian Qian, Song Yuan, Shaohua Wang, Guoling Zhou, Deborah Baofeng Wang, Minjie Ye, Zhenyu Hu, Haihang Yu

**Affiliations:** 1 Affiliated Kangning Hospital of Ningbo University, Ningbo, Zhejiang, China; 2 School of Medicine, Ningbo University, Ningbo, Zhejiang, China; 3 Quzhou Third Hospital, Quzhou, Zhejiang, China; 4 Zhoushan Second People’s Hospital, Zhoushan, Zhejiang, China; 5 Affiliated Mental Health Center & Hangzhou Seventh People’s Hospital, Zhejiang University School of Medicine, Hangzhou, Zhejiang, China; 6 Zhejiang Provincial Clinical Research Center for Mental Disorders, the Affiliated Wenzhou Kangning Hospital, Wenzhou Medical University, Wenzhou, Zhejiang, China

**Keywords:** adolescent, anxiety, primary schools, school phobia, secondary schools, sex difference

## Abstract

**Background:**

Increasing number of outpatient visits has been related to mental health crisis in adolescents. However, little is known for the chronological changes in adolescent anxiety symptoms in community samples, especially in China.

**Methods:**

Students from the 4th to the 11th grade were recruited from representative local schools in Zhejiang province. Screen for Child Anxiety-Related Emotional Disorders (SCARED) were used to collect the scores of panic anxiety, generalized anxiety, separation anxiety, social anxiety and school phobia, and were compared with the records of 2001.

**Results:**

Data from 8287 students showed that the anxiety symptoms were more frequent in girls and in higher grades. Chronological comparison suggested that all sub-types of anxiety have increased in girls, and symptoms of school phobia have increased in boys in middle schools. Largest size-effect of increase was seen for school anxiety.

**Conclusion:**

This study demonstrates a substantial increase in anxiety symptoms among Chinese adolescents over time, particularly among girls, with school-related anxiety showing the most pronounced rise. These findings indicate a growing mental health burden in community-based adolescent populations and underscore the importance of early identification and school-based preventive strategies.

## Introduction

Anxiety disorders include disorders that share features of excessive fear and anxiety and related behavioral disturbances. Several types of anxiety could be developed across life span. Whereas separation anxiety often onsets in childhood, social anxiety, panic disorder, and generalized anxiety typically develop in adolescents. Population-based epidemiological studies indicate that anxiety disorder is the most common mental disorder among children and adolescents, and its first onset usually occurs in childhood, much earlier than the onset of depression or other mental disorders (1). Anxiety symptoms in adolescents not only closely relates to depressive mood and suicidal ideation (2), but also predicts later mental health problems (3).

The increment of adolescent distress has begun to be recognized in recent years in both the United States and the United Kingdom (4). Repeated cross-sectional and cohort studies have documented a substantial increase in anxiety prevalence over time, with one large school-based survey reporting that the proportion of adolescents meeting anxiety screening criteria rose from 34.1% in 2012 to 44.0% in 2018 (5). Notably, these increases were more pronounced among girls (5). However, evidence regarding adolescent anxiety in China remains limited. Most existing research has relied on cross-sectional designs or clinical samples, providing little information on how adolescent anxiety has evolved over time.

In this survey, we reported the profile of anxiety in local primary, middle, and high schools in Zhejiang Province in China, and compared it with the historical records acquired twenty years ago. By examining anxiety domains across sexes and school stages in a community-based sample, this study aims to provide empirical evidence on temporal changes in adolescent anxiety and to inform future research, early identification, and school-based prevention efforts.

## Methods

### Participants

Participants were from twenty-four representative schools of five cities in Zhejiang Province during September 2021 to April 2023. Using a stratified cluster sampling method, 9 schools were selected from each prefecture-level city (3 primary schools, 3 middle schools, and 3 high schools) to cover both urban and rural areas. In every grade in the schools, 2 classes were chosen randomly. Once chosen, the whole class of students were inspected. Students ranged from the 4th grade to the 11th grade, aged 10 to 18 years old. Students in the 9th grade were not recruited since the coming examination of high school entrance was well considered as a critical life event, and the research group did not want to introduce any kind of mind disturbance that could be related to the survey activity. The study protocol was approved by the Ethic Committee of the Affiliated Kangning Hospital of Ningbo University (NBKNYY-2020-LC-52). Informed consents were given by students and their parents. For 8,432 answers included in the survey, 110 were removed for the straight-line answering style, and 35 removed for incompletion of the critical questionnaires, leading to a final number of 8287.

### Questionnaires

The questionnaires including brief school and family environment investigation, The Screen for Child Anxiety Related Emotional Disorders (SCARED), Center of Epidemiological Studies- Depression (CES-D) Scale, and the Parental Bonding Instrument (PBI). The sheet can be finished within 30 minutes for a grade 4 student.

The Screen for Child Anxiety-Related Emotional Disorders (SCARED), a self-report screening instrument designed to measure anxiety disorder symptom dimensions in children and adolescents. It consists of 41 items assessing five symptom dimensions: Generalized Anxiety Disorder (9 items), Panic Disorder (13 items), Separation Anxiety Disorder (8 items), Social Anxiety Disorder (4 items), and School Anxiety (or School Refusal; 4 items). With the exception of the School Anxiety dimension, the remaining four dimensions correspond closely to DSM-5 anxiety disorder symptom domains. Participants rated each item on a three-point Likert scale ranging from 0 (almost never) to 2 (often), and domain scores were calculated by summing the corresponding item scores, with higher scores indicating greater anxiety symptom severity rather than clinical diagnoses. The possible score ranges were 0–18 for Generalized Anxiety Disorder, 0–26 for Panic Disorder, 0–16 for Separation Anxiety Disorder, and 0–8 for both Social Anxiety Disorder and School Anxiety. Previous studies have demonstrated good internal consistency for the SCARED domains, with Cronbach’s alpha coefficients ranging from 0.84 to 0.87 for Generalized Anxiety Disorder, 0.83 to 0.90 for Panic Disorder, 0.68 to 0.79 for Separation Anxiety Disorder, and 0.80 to 0.86 for Social Anxiety Disorder (6).

The Center for Epidemiologic Studies Depression Scale (CES-D), a widely used self-report instrument designed to measure the frequency and severity of depressive symptoms in community populations. It consists of 20 items rated on a four-point Likert scale reflecting symptom frequency over the past week, with response options ranging from 0 (rarely or none of the time) to 3 (most or all of the time). Total CES-D scores were calculated by summing all item scores, with higher scores indicating greater depressive symptom severity. The scale conceptualizes depression as a multidimensional construct encompassing depressed affect, positive affect, somatic complaints, and interpersonal difficulties. Previous studies have demonstrated acceptable to good internal consistency of the CES-D among adolescents, with Cronbach’s alpha coefficients typically exceeding 0.80 (7).

### Statistic analysis

Data were processed with python 3.8. Those with coding errors or answers that were not included in options were re-coded as missing values. Multivariable linear regression model was used to estimate the effects of sex and grade to the anxiety symptoms, with sex as a categorical variable, and grade as a continuous variable. To examine associations of sex and grade with anxiety symptoms, multivariable linear regression models were fitted for each outcome (sub-scales and total score), with sex entered as a categorical variable and grade treated as a continuous variable. For group comparisons by sex and age group (**Table 1**), subscale and total scores were compared across four sex-by-age categories using one-way analysis of variance (ANOVA). Historical records extracted from Wang et al (8) were compared with sex- and age- matched current samples using 2-sample t-test. Cohen’s d was calculated for each historical-current pair. High school students were removed from comparison because there had been no records from high school students. A two-tailed p value less than 0.05 was considered as statistical significant. To control for multiple testing across outcomes (five subscales plus the total score), p values within each family of comparisons were adjusted using the Holm–Bonferroni procedure, and corrected significance thresholds were applied. All tests were two-tailed, and a corrected p value < 0.05 was considered statistically significant.

**Table 1:** Comparison of the sub-scales between different gender and age groups.

Item	Boy		Girl		F	Comparison between different groups
	9 to 12 age (n=1675)	13 to 16 age (n=2375)	9 to 12 age (n=1594)	13 to 16 age (n=1919)		
somatic/panic	3.81 ± 4.60	3.83 ± 4.85	3.70 ± 4.68	3.93 ± 4.85	0.68	n.s.
general anxiety	4.27 ± 3.88	4.24 ± 4.03	4.08 ± 3.83	4.36 ± 4.06	1.44	4 > 3
separation anxiety	3.16 ± 2.84	3.31 ± 3.03	3.17 ± 2.93	3.31 ± 3.05	1.49	n.s.
social anxiety	4.39 ± 3.62	4.63 ± 3.62	4.20 ± 3.53	4.52 ± 3.62	4.85^b^	2 > 1, 3; 4 > 3
school anxiety	1.39 ± 1.73	1.46 ± 1.80	1.27 ± 1.67	1.50 ± 1.81	5.56^b^	2, 4 > 3
Total	17.01 ± 13.65	17.46 ± 14.31	16.42 ± 13.71	17.61 ± 14.61	2.52	2, 4 > 3

P < 0. 05, ^b^ P < 0. 01, n.s.= not significant (p ≥ 0.05).

## Results

The analysis was conducted on 3,014 pupils in primary school, 2,446 students in middle school, and 2827 students in high school, with 46.6% female.

Scores of anxiety symptoms were summarized in **Table 2**. Generally, anxiety symptoms were more frequent in girls and in higher grades. Looking into the sub-types of anxiety, it could be found that generalized anxiety, social anxiety, and school phobia increased, while the separation anxiety decreased, with the grades. Interaction effects between sex and grades were significant expect for Panic symptoms and social anxiety.

**Table 2:** SCARED sub-scales and total scores in students from 4^th^ to 11^th^ grades.

Grade	sex	N	SCARED					
			Total scores	Panic/somatic	Generalized anxiety	Separation anxiety	Social anxiety	School phobia
4th	Male	593	14.5 ± 12.7	3.35 ± 4.44	3.31 ± 3.41	3.57 ± 3.05	3.47 ± 3.06	0.85 ± 1.33
	female	521	17.06 ± 13.6	3.78 ± 4.67	3.70 ± 3.58	4.32 ± 3.52	4.26 ± 3.25	0.99 ± 1.48
5th	male	592	13.8 ± 11.8	3.06 ± 3.84	3.15 ± 3.18	3.31 ± 2.93	3.33 ± 3.07	0.92 ± 1.45
	female	608	16.9 ± 12.9	3.97 ± 4.61	3.83 ± 3.51	3.82 ± 3.14	4.17 ± 3.11	1.13 ± 1.52
6th	male	374	14.2 ± 12.1	3.25 ± 4.25	3.40 ± 3.41	2.96 ± 2.80	3.61 ± 3.08	0.98 ± 1.42
	female	325	19.5 ± 13.1	4.68 ± 4.95	4.80 ± 3.70	3.91 ± 2.88	4.74 ± 3.25	1.34 ± 1.64
7th	male	715	14.6 ± 14.8	3.54 ± 5.08	3.59 ± 3.95	2.80 ± 2.98	3.57 ± 3.51	1.08 ± 1.63
	female	607	18.7 ± 15.2	4.56 ± 5.47	4.55 ± 4.17	3.57 ± 3.09	4.48 ± 3.71	1.49 ± 1.77
8th	male	575	15.4 ± 15.7	3.72 ± 5.32	3.77 ± 4.21	2.76 ± 3.01	3.91 ± 3.86	1.23 ± 1.66
	female	549	21.1 ± 15.2	4.81 ± 5.01	5.37 ± 4.30	3.81 ± 3.14	5.20 ± 3.71	1.95 ± 1.98
10th	male	931	15.9 ± 13.3	3.27 ± 4.33	3.98 ± 3.84	2.41 ± 2.51	4.71 ± 3.69	1.52 ± 1.75
	female	750	22.8 ± 14.7	4.77 ± 5.10	6.10 ± 4.22	3.86 ± 3.08	5.89 ± 3.65	2.22 ± 2.06
11th	male	645	17.5 ± 13.7	3.41 ± 4.18	4.45 ± 3.88	2.64 ± 2.60	5.34 ± 3.74	1.69 ± 1.93
	female	499	23.4 ± 13.9	4.75 ± 4.95	6.19 ± 4.10	3.86 ± 2.84	6.35 ± 3.69	2.24 ± 1.89
The F (p values) of the multivariable linear regression model for each sub-scales								
Grade			120.7 (<10-6)	11.1 (<10-6)	230.8 (<10-6)	48.5 (<10-6)	338.1 (<10-6)	405.4 (<10-6)
Sex			254.4 (<10-6)	112.1 (<10-6)	237.1 (<10-6)	226.0 (<10-6)	174.5 (<10-6)	149.9 (<10-6)
Grade * sex			20.6 (<10-6)	7.7 (0.0564)	41.4 (<10-6)	16.9 (<10-6)	1.92 (0.166)	22.9 (<10-6)

The distribution of subscale scores across age groups in boys and girls is summarized in **Table 1**. Overall, adolescents aged 13–16 years tended to report higher levels of anxiety-related symptoms than those aged 9–12 years, with the age-related increase being most evident for social anxiety, school anxiety, and the total score. In contrast, somatic/panic symptoms and separation anxiety showed no clear age-related differences. With respect to gender patterns, girls generally exhibited higher symptom levels than boys, particularly in the older age group. Among adolescents aged 13–16 years, girls showed the highest scores on general anxiety, social anxiety, and the total score compared with the other groups. In the younger age group (9–12 years), boys and girls displayed broadly comparable levels on most sub-scales, although modest sex-related differences could still be observed in specific symptom domains. Notably, school anxiety appeared to vary primarily with age rather than sex, with no consistent gender disparity across age groups.

**Figure 1:**
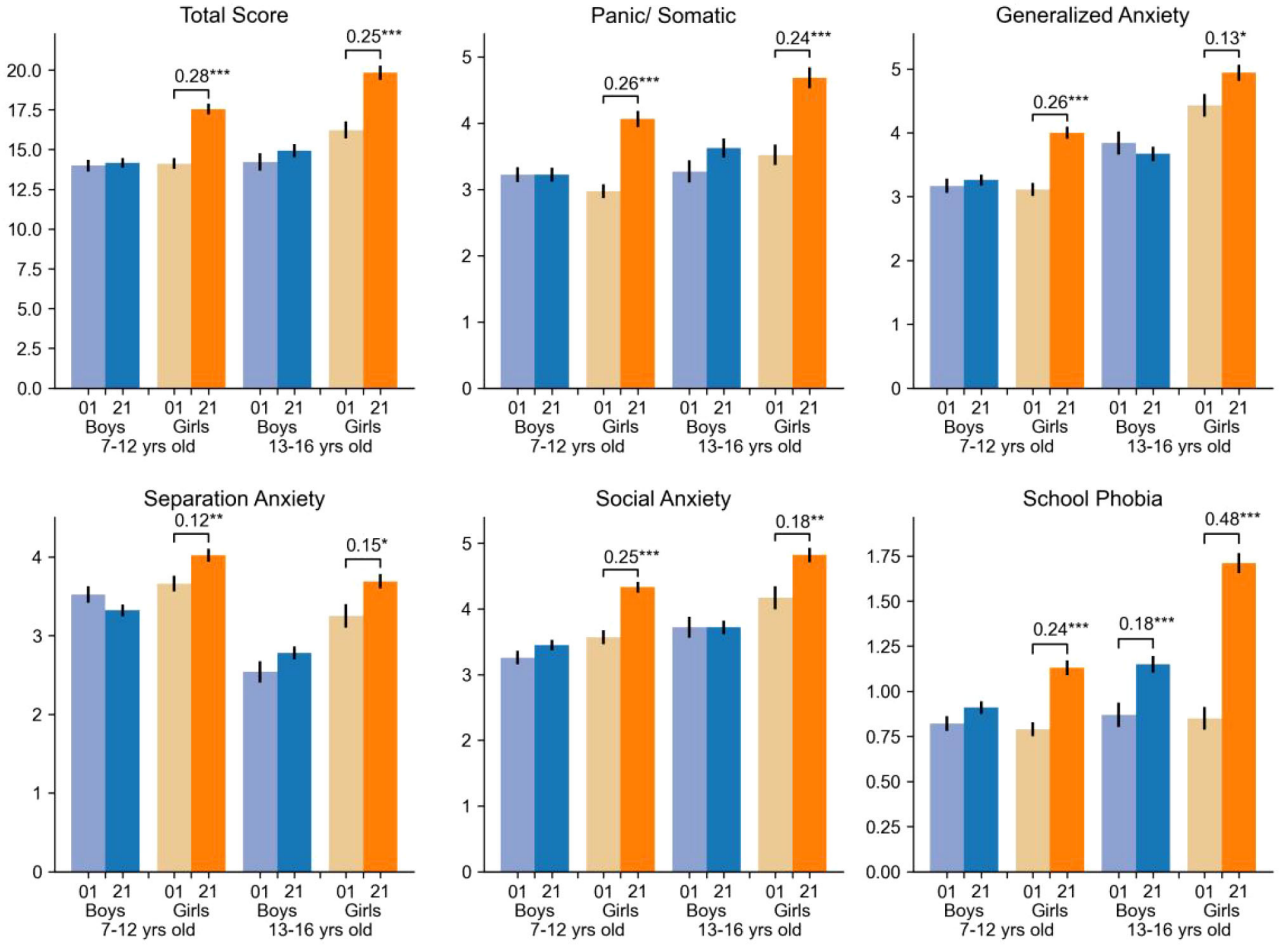
Temporal comparison of anxiety total and subscale scores (current sample vs. 2001 cohort) stratified by sex and age.

Compared with the records in 2001 (**Figure 1**), boys had insignificant difference in most of the anxiety sub-types. The only increase in boys was school phobia in middle school. For girls, all facets of the anxiety symptoms had increased both in primary and middle schools. The largest size-effect was seen for school anxiety in girls in middle school.

## Discussion

In this study, three main findings emerged. Firstly, compared to historical data, the anxiety index among Chinese teenagers in the current sample is higher. Secondly, compared to males, anxiety symptoms are more pronounced in adolescent females. Third, among the anxiety dimensions assessed, school phobia is the most prominent sub-type.

The finding of this study that the current level of anxiety symptoms among adolescents is higher than in the past is generally consistent with significant research results on the burden of adolescent anxiety in other countries. For instance, a study in the United States on trends and differences in adolescent anxiety over time also showed that the prevalence of adolescents meeting the anxiety screening criteria increased from 34.1% in 2012 to 44% in 2018 (5). In addition, an analysis of trends in adolescent mental health in Canada also reflects this trend (9). However, we cannot ignore the potential impact that over twenty years of changes in education and social environment may have had. A cohort study in Shanghai, China in 2023 showed that among students in grades 6 to 9, excessive homework burden may lead to neurobehavioral problems, and this association is more pronounced in girls. Sleep deprivation may mediate this association in a gender-specific manner (10). A 2014 epidemiological survey showed that 51.0% of adolescents did not get the ideal amount of sleep (11). In a 2023 study, the prevalence of insufficient sleep and late bedtimes among adolescents in grades 6–9 reached 44.0%-55.0% and 40.3%-91.6%, respectively (10). In addition, the use of social media has permeated all aspects of the lives of children and adolescents, and the rapid expansion of digital media and social networking platforms has brought new sources of stress to teenagers. A cohort study in the United States involving 11,876 children and adolescents found that individuals with above-average social media use during the first and second year after the baseline survey were more likely to exhibit depressive symptoms in the following year (12). Another study in the UK also confirmed that problematic social media use is associated with depressive and anxiety symptoms in children and adolescents (13). Similar phenomena have been observed in China, where adolescents’ lifestyle patterns have shifted towards higher levels of screen-related sedentary behavior. For example, a study in underdeveloped regions of China showed that students who used mobile phones or computers for ≤2 hours per day were significantly less likely to experience severe anxiety compared to those who used mobile phones or computers for >2 hours per day (14).

In terms of gender differences, our research findings are also consistent with existing literature. Longitudinal cohort studies in Europe suggest that even after accounting for familial, psychosocial, and biological factors, being female is the only significant predictor of anxiety disorders and is also the strongest predictor of their onset (15).

In terms of the anxiety dimension, the results of this study emphasize school phobia, whereas international research findings are more diverse. International research indicates that the distribution and development of anxiety sub-types need to take into account the influence of different age stages and sociocultural backgrounds. A community study in Norway showed that specific phobias are the most common anxiety disorders in early and middle childhood, while the prevalence of generalized anxiety disorder increases during adolescence (16). Community studies from India and Australia reported that social anxiety disorder and specific phobias are among the most common anxiety disorders in teenagers (17, 18). A longitudinal study conducted in Portugal showed that academic performance, learning disabilities, and externalizing symptoms are the strongest predictors of anxiety levels (19), which also suggests that our findings are not an isolated phenomenon.

As a cross-sectional study, this work has several limitations when comparing the current data with the historical dataset from 2001, and it does not allow causal or longitudinal inferences. First, the present sample covers Grades 4–8 and does not include students in Grades 2–3 or Grade 9, whereas the 2001 reference study included Grades 2–9 and employed an equal-per-grade sampling design. Because anxiety symptoms may change nonlinearly across development and because different grades can differ in both mean symptom levels and variance structures, the inconsistency in grade coverage may introduce grade-composition bias. In addition, ninth grade constitutes a high-stakes examination stage in the education system and may be accompanied by greater academic pressure and emotional fluctuations; therefore, our sample did not capture a potentially higher-stress grade included in the reference cohort, further weakening the strength of any inference regarding temporal trends.

## Conclusions

Nonetheless, this study still holds significant implications for research and practice in adolescent mental health. Based on a large community sample and using the same assessment tools, it provides a comparison with historical conditions. The identification of school phobia, a particularly prominent area of anxiety, highlights that for Chinese adolescents currently, the school environment is one of the important settings for early identification and prevention efforts. From a public health and educational perspective, these findings emphasize the potential value of school-based mental health screening and targeted interventions aimed at alleviating school-related distress. Additionally, the observed gender differences suggest that prevention strategies may benefit from being sensitive to gender-specific vulnerabilities.

## Data Availability

The original contributions presented in the study are included in the article/Supplementary Material. Further inquiries can be directed to the corresponding authors.

## References

[1] Wehry AM Beesdo-Baum K Hennelly MM Connolly SD Strawn JR . Assessment and treatment of anxiety disorders in children and adolescents. Curr Psychiatry Rep. (2015) 17:52. doi: 10.1007/s11920-015-0591-z, PMID: 25980507 PMC4480225

[2] Zou S Song X Tan W Deng F Zhang H Xu H . Core self-evaluation as mediator between depressive symptoms and suicidal ideation in adolescents. J Affect Disord. (2022) 302:361–6. doi: 10.1016/j.jad.2022.01.093, PMID: 35104465

[3] Mulraney M Coghill D Bishop C Mehmed Y Sciberras E Sawyer M . A systematic review of the persistence of childhood mental health problems into adulthood. Neurosci Biobehav Rev. (2021) 129:182–205. doi: 10.1016/j.neubiorev.2021.07.030, PMID: 34363845

[4] Blanchflower DG Bryson A Xu X . The declining mental health of the young and the global disappearance of the unhappiness hump shape in age. PloS One. (2025) 20:e0327858. doi: 10.1371/journal.pone.0327858, PMID: 40864616 PMC12385385

[5] Parodi KB Holt MK Green JG Porche MV Koenig B Xuan Z . Time trends and disparities in anxiety among adolescents, 2012–2018. Soc Psychiatry Psychiatr Epidemiol. (2022) 57:127–37. doi: 10.1007/s00127-021-02122-9, PMID: 34100110 PMC8183580

[6] Hale Iii WW Raaijmakers QA Van Hoof A . Improving screening cut-off scores for DSM-5 adolescent anxiety disorder symptom dimensions with the screen for child anxiety related emotional disorders. Psychiatry J. (2014) 2014:517527. doi: 10.1155/2014/517527, PMID: 24829901 PMC3994902

[7] Blodgett JM Lachance CC Stubbs B Co M Wu YT Prina M . A systematic review of the latent structure of the Center for Epidemiologic Studies Depression Scale (CES-D) amongst adolescents. BMC Psychiatry. (2021) 21:197. doi: 10.1186/s12888-021-03206-1, PMID: 33874939 PMC8054366

[8] Shen W Zhang W Ye M Tan S Yuan S Wang S . Profile of anxiety symptoms in adolescents in Zhejiang, China. J Affect Disord. (2026) 395:120787. doi: 10.1016/j.jad.2025.120787, PMID: 41325810

[9] Wiens K Bhattarai A Pedram P Dores A Williams J Bulloch A . A growing need for youth mental health services in Canada: examining trends in youth mental health from 2011 to 2018. Epidemiol Psychiatr Sci. (2020) 29:e115. doi: 10.1017/S2045796020000281, PMID: 32299531 PMC7214527

[10] Yu T Xu D Fan J Hua H Guo X Zhang Y . Homework, sleep insufficiency and adolescent neurobehavioral problems: Shanghai Adolescent Cohort. J Affect Disord. (2023) 332:273–82. doi: 10.1016/j.jad.2023.04.008, PMID: 37059191

[11] Chen T Wu Z Shen Z Zhang J Shen X Li S . Sleep duration in Chinese adolescents: biological, environmental, and behavioral predictors. Sleep Med. (2014) 15:1345–53. doi: 10.1016/j.sleep.2014.05.018, PMID: 25277663

[12] Nagata JM Otmar CD Shim J Balasubramanian P Cheng CM Li EJ . Social media use and depressive symptoms during early adolescence. JAMA Netw Open. (2025) 8:e2511704. doi: 10.1001/jamanetworkopen.2025.11704, PMID: 40397441 PMC12096259

[13] Saleem N Young P Yousuf S . Exploring the relationship between social media use and symptoms of depression and anxiety among children and adolescents: A systematic narrative review. Cyberpsychol Behav Soc Netw. (2024) 27:771–97. doi: 10.1089/cyber.2023.0456, PMID: 39446668

[14] Wen X Zhu F Yuan Z Mao Z . Relationship between physical activity, screen-related sedentary behaviors and anxiety among adolescents in less developed areas of China. Medicine. (2022) 101:e30848. doi: 10.1097/MD.0000000000030848, PMID: 36181048 PMC9524945

[15] Narmandakh A Roest AM De Jonge P Oldehinkel AJ . Psychosocial and biological risk factors of anxiety disorders in adolescents: a TRAILS report. Eur Child Adolesc Psychiatry. (2021) 30:1969–82. doi: 10.1007/s00787-020-01669-3, PMID: 33113027 PMC8563629

[16] Steinsbekk S Ranum B Wichstrøm L . Prevalence and course of anxiety disorders and symptoms from preschool to adolescence: a 6-wave community study. J Child Psychol Psychiatry Allied Discip. (2022) 63:527–34. doi: 10.1111/jcpp.13487, PMID: 34318492

[17] Madasu S Malhotra S Kant S Sagar R Mishra AK Misra P . Prevalence and determinants of anxiety disorders among adolescents in a rural community from northern India. Asian J Psychiatry. (2019) 43:137–42. doi: 10.1016/j.ajp.2019.05.009, PMID: 31146170

[18] Spence SH Zubrick SR Lawrence D . A profile of social, separation and generalized anxiety disorders in an Australian nationally representative sample of children and adolescents: Prevalence, comorbidity and correlates. Aust New Z J Psychiatry. (2018) 52:446–60. doi: 10.1177/0004867417741981, PMID: 29185356

[19] Loparo D Fonseca AC Matos APM Craighead WE . Anxiety and depression from childhood to young adulthood: trajectories and risk factors. Child Psychiatry Hum Dev. (2024) 55:127–36. doi: 10.1007/s10578-022-01391-y, PMID: 35763175

